# Effect of melatonin/BMP-2 co-delivery scaffolds on the osteoclast activity

**DOI:** 10.1007/s10856-021-06502-0

**Published:** 2021-03-22

**Authors:** Hala Jarrar, Damla Çetin Altındal, Menemşe Gümüşderelioğlu

**Affiliations:** Bioengineering Department, Hacettepe University, 06800 Beytepe, Ankara Turkey

## Abstract

Bone morphogenetic protein two (BMP-2) has been widely used as an osteoinductive agent in the treatment of bone diseases. However, some side effects, such as osteoclast activation have emerged when it was used at high doses. In this study, by considering the osteoclast-suppressing capability of melatonin (MEL), its effect on osteoclast differentiation induced by BMP-2 was investigated. These two factors, MEL and BMP-2, were embedded into chitosan/hydroxyapatite (HAp) scaffolds that were characterized morphologically by scanning electron microscopy (SEM) and micro-computed tomography (μ-CT). Release profiles of MEL and BMP-2 from scaffolds were determined in vitro and then, the differentiation of RAW 264.7 cells to osteoclasts was investigated on the scaffolds. Results of tartrate-resistant acid phosphatase (TRAP) staining, SEM imaging and expression of cathepsin K gene showed that, in the presence of BMP-2, osteoclast differentiation increased, whereas it decreased in MEL and MEL/BMP-2 embedded scaffolds suggesting that melatonin successfully attenuated osteoclast differentiation induced by BMP-2. Thus, the MEL/BMP-2 loaded chitosan/HAp scaffolds that have dual function in enhancing bone formation and inhibiting osteoclast activity are recommended biomaterials in the field of bone regeneration.

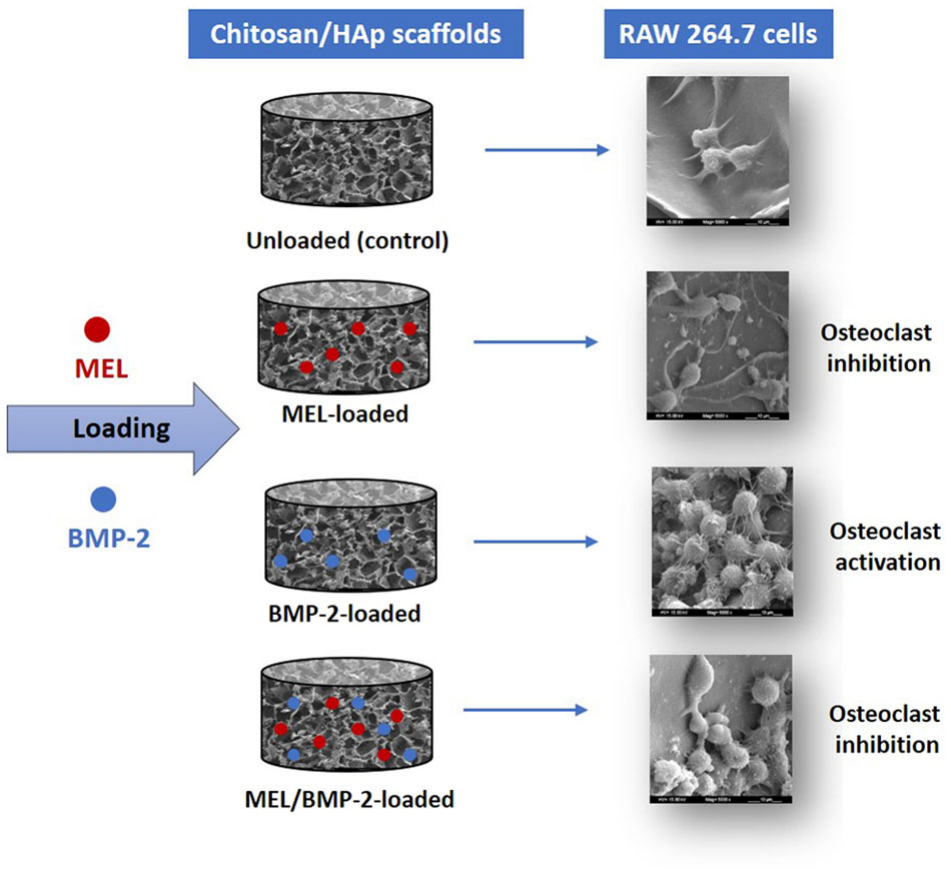

## Introduction

Bone morphogenetic proteins (BMPs) belong to transforming growth factors (GFs) beta superfamily which else includes activins, inhibins and growth differentiation factors [1]. Among BMPs, BMP-2 is the most studied one and known to be a strong osteoinductive agent. The osteogenic activity of BMP-2 has been investigated clinically and resulted in the commercialization of BMP-2-based bone substitutes approved by Food and Drug Administration as an alternative of autologous grafts [2, 3]. However, by increasing BMP-2 use in the treatment of bone diseases, many side effects have emerged. The adverse side effects were found to be associated with the use of BMP-2 at high doses and those include; inflammation, ectopic bone formation and osteoclast activation [4–7]. Since osteoclasts play a critical role in bone repair and homeostasis, the effect of BMP-2 on osteoclast activation was elucidated in many in vitro studies. For instance, it was reported that BMP-2 directly enhanced suboptimal receptor activator of nuclear factor kappa-Β ligand (RANKL)-stimulated differentiation of bone marrow-derived cells to osteoclasts [8]. Also, BMP-2 was found to stimulate the differentiation of these cells in the presence of RANKL and macrophage stimulating factor (MCSF) [9]. Concurrently with the elevation of complications caused by the use of BMP-2 high doses, the use of appropriate doses, carriers and implementation of supplemental factors have been brought to light to improve BMP-2 use [10]. In one of the studies, a dual delivery system of placental growth factor-2 (PIGF) and BMP-2 was developed using heparin-based nanocomplex to deliver both of these osteoinductive factors at minimal concentrations over an extended time period [11]. The dual delivery of GFs improved the differentiation of MC3T3-E1 cells toward osteoblast comparing to single delivery systems and results suggested that the activity of BMP-2 may be amplified by combining it with PIGF within a delivery system. BMP-2 was also combined with alendronate which has both osteoinductive and osteoclast-suppressing properties in order to evaluate the effect of alendronate on BMP-2 high dose-mediated osteoclast activation. It was reported that osteoclast activation in defect area containing collagen sponges loaded with the two factors, alendronate and BMP-2, was lower than that of BMP-2-loaded group indicating that alendronate suppressed resorption caused by BMP-2 high dose [12].

Melatonin (MEL) (N-acetyl-5-methoxytryptamine), which is mainly produced in pineal gland, does not only regulate sleep/wake cycle and circadian rhythm and provide antioxidant protection and anticancer activity, but also plays a role in bone homeostasis [13]. Recent studies have demonstrated that MEL enhances osteoblast differentiation through activation of BMP/ERK/Wnt signaling pathways, meanwhile, it inhibits osteoclast differentiation by increasing the expression of osteoprotegerin in osteoblasts which is considered as an antagonistic agent for RANKL [14, 15]. MEL also inhibits osteoclast differentiation directly by downregulation of RANKL-induced NF-ĸB pathways [16].

In the present work, we hypothesized that MEL embedding into BMP-2-loaded chitosan/HAp scaffolds would alleviate BMP-2-mediated osteoclast differentiation. Differentiation behavior of osteoclasts was studied by seeding RAW 264.7 cells into MEL-loaded and/or BMP-2-loaded chitosan/HAp scaffolds.

## Materials and methods

### Materials

Chitosan (deacetylation degree: ≥92.6%) and acetic acid were obtained from Heppe MEDICAL CHITOSAN GmbH (USA) and Riedel de Haen (Germany), respectively. Ethanol was purchased from Sigma (Germany) and hydroxyapatite (HAp) in bead form (55–110 μm) was obtained from Science Application Industries (France). Phosphate buffer saline (PBS) tablets and sodium azide were bought from Sigma-Aldrich (Germany). Recombinant BMP-2 and BMP-2 ELISA kit were obtained from Thermo Fisher Scientific (USA) and MEL was obtained from Sigma-Aldrich (Germany). For cell culture studies, RAW 264.7 cell line (ATCC^®^ TIB-71™) used in was purchased from American Type Culture Collection (ATCC). RANKL, MCSF, MTT (3-[4,5-dimethylthiazol-2-yl]-diphenyltetrazolium bromide) and isopropanol were all bought from Sigma-Aldrich (Germany). Dulbecco’s Modified Eagle Medium (DMEM-high glucose), fetal bovine serum (FBS), penicillin-streptomycin, l-glutamine and Dulbecco’s phosphate buffer saline were obtained from Capricorn Scientific (Germany). Glutaraldehyde, hexamethyldisilazane (HMDS), p-nitrophenyl phosphate (pNPP) tablets, iron chloride hexahydrate, naphthol AS-MX phosphate and fast red violet LB salt were all purchased from Sigma (Germany). Disodium tartrate dehydrate and N, N-dimethylformamide were bought from Merck (Germany). Sodium acetate anhydrous was obtained from Riedel de Haen (Germany).

### Fabrication and characterization of chitosan/HAp scaffolds

Chitosan/HAp scaffolds were fabricated by freeze-drying method [17]. In brief, chitosan solution with concentration of 2% (w/v) was prepared by dissolving 2 g chitosan in 100 mL distilled water containing 1 mL acetic acid. Chitosan solution was mixed overnight by magnetic stirrer until clear solution obtained. Then, 1.5% (w/v) HAp beads were added to chitosan solution and mixed for 3 h. Final solution was poured into 24-well tissue culture polystyrene plates (TCPS) to a thickness of about 0.5 cm. Plates were first frozen for 2 days at −20 °C, then lyophilized for other 2 days at −80 °C in a freeze-drier (Christ, Germany) to obtain the final porous chitosan scaffolds. For scaffolds stabilization, lyophilized scaffolds were soaked in 96% ethanol for 2 days and then, in 70% ethanol for 1 h.

#### Scanning electron microscopy (SEM)

The surface morphology of scaffolds, interconnected structure and the distribution of HAp beads within chitosan scaffolds were observed by SEM (Zeiss, Evo-50, Germany). The pore size of the scaffold was measured from the SEM images with ImageJ software (NIH, Bethesda, MD, USA) by calculating the average of randomly selected pores.

#### Micro-computed tomography (μ-CT)

The porous and interconnected structure of the scaffolds was observed by images obtained by μ-CT device (Bruker, Skyscan 1272, Germany). The µ-CT analyzer software (CTAn, version 1.13 SkyScan, Bruker, Belgium) was used to calculate the porosity and closed pore percentages.

#### Mechanical analysis

The dried scaffolds were cut into cylindrical shapes of specific dimensions (9 mm diameter, 2 mm height) for mechanical testing using Texture Analyzer (TA.XT Plus, UK). The samples were swollen in PBS (pH: 7.4) at 37 °C before testing.

#### Thermogravimetric analysis

Thermogravimetric analysis was carried out at 10 °C/min heating rate in the range of 25–600 °C in nitrogen atmosphere by using TG/DTA 6300 SII EXSTAR 6000 (Seiko Instruments Inc., USA).

### MEL and BMP-2 loading and their in vitro release from chitosan/HAp scaffolds

Incorporation of BMP-2 and MEL into chitosan/HAp scaffolds was performed by embedding technique. In this technique, BMP-2 and MEL solutions were pipetted onto the scaffolds. Loading amounts of BMP-2 and MEL were determined according to the literature and our previous studies, respectively. Our previous study showed that a dose of 800 µM MEL had the best inhibitory effect on the differentiation of RAW 264.7 cells into osteoclasts when the inoculation density was 1 × 10^4^ cells/mL [18]. This is why, 300 ng BMP-2 and 186 μg MEL (leads to 800 µM) were embedded, respectively, by the addition of 40 μL of BMP-2 or MEL solution drop by drop with a micropipette into each scaffold. Embedded scaffolds were then dried overnight by freeze dryer.

To obtain release profiles of MEL-loaded and BMP-2-loaded chitosan/HAp scaffolds, they were immersed into 1 mL PBS containing 0.1% (w/v) sodium azide (pH: 7.4) in three different parallels and incubated with gentle shaking (70 rpm) at 37 °C for 7 days. At selected time intervals, samples were collected and replaced with fresh amounts of PBS to maintain sink condition. Absorbance of MEL in supernatant was measured by Nanodrop 2000 (Thermo Scientific, USA) at 279 nm and subsequently the amount of MEL was calculated by using calibration curve using the standard samples (*y* = 2.7876x + 0.0123, *R*^2^ = 0.9959; y: absorbance, x: concentration of MEL in PBS, mg/mL). Meanwhile, the amount of BMP-2 present in supernatant was measured by BMP-2 ELISA kit according to the manufacturers’ instructions.

### Cell culture studies

Cell culture studies were performed with osteoclast precursor RAW 264.7 cells. RAW 264.7 is monocyte/macrophage cell line derived from Abelson murine leukemia virus-induced tumor in *Mus musculus* mice. RAW 264.7 cells have the capacity to differentiate toward osteoclasts in the existence of RANKL. Prior to cell seeding, chitosan/HAp scaffolds were sterilized, and then loaded with MEL and/or BMP-2. For sterilization process, scaffolds having diameter of 9 mm and thickness of 2 mm were immersed into 70% ethanol for 30 min, washed thrice with DPBS and exposed to UV irradiation (30 min for each surface). Then, scaffolds were conditioned with DMEM-high glucose supplemented with 10% (v/v) FBS, 2% (v/v) l-glutamine, 1% (v/v) penicillin/streptomycin. After the removal of medium, scaffolds were maintained overnight in CO_2_ incubator to obtain more dehydrated scaffolds.

Four different scaffolds were involved in this experiment: blank scaffolds, MEL-loaded scaffolds, BMP-2-loaded scaffolds and MEL/BMP-2-loaded scaffolds. Embedding of MEL and BMP-2 into scaffolds was carried out by adding a final volume of 40 µL of drug solution to reach final concentrations of 300 ng/mL of BMP-2 and 800 µM of MEL while blank scaffolds were prepared by replacing the volume by sterile ultra-pure water. Embedded scaffolds were then left in laminar cabinet for 2 h to achieve relative dehydration.

RAW 264.7 cells were harvested from the flasks by cell scraper. Cell suspension was added to each scaffold (30 µL) to maintain 5 × 10^5^ cells/scaffold inoculation density for each scaffold. After 3 h of cell seeding, 1 mL osteoclastogenic medium (proliferation medium supplemented with 10 ng/mL RANKL and 10 ng/mL MCSF) was added to each well and incubated in 5% CO_2_ atmosphere at 37 °C. The medium was replenished every 2 days.

#### Cell viability

Mitochondrial activities of osteoclast precursors (RAW 264.7 cells) on MEL and BMP-2-loaded chitosan/HAp scaffolds were assessed by MTT at different culture times up to 8 days. At selected times, the culture medium was aspirated and scaffolds were transferred to another 24-well TCPS. Then, 600 μL pre-warmed culture medium supplemented with 60 μL MTT solution (2.5 mg/mL MTT dissolved in PBS) was added to each sample, which was then incubated at 37 °C for 3 h. After the incubation, 400 μL of 0.04 M HCl containing isopropanol was added to each well to dissolve the purple formazan crystals. Optical densities of 200 μL of the resulting solutions were measured spectrophotometrically at 570 nm with reference to 690 nm using a microplate reader (ASYS, Hitech UVM 340 plate reader, Austria).

#### Tartrate-resistant acid phosphatase (TRAP) assay and TRAP staining

TRAP staining and TRAP activity were performed to evaluate the differentiation of RAW 264.7 cells cultured on blank and MEL and/or BMP-2-embedded chitosan/HAp scaffolds. In order to visualize TRAP+ cells seeded on chitosan/HAp scaffolds, scaffolds were transferred to new 24-well TCPS after 8 days of the culture. Samples were fixed with 4% (v/v) formaldehyde for 10 min and subsequently with 1:1 (v/v) acetone/ethanol solution for 1 min [19]. Then, cells were incubated for 30 min in staining solution (0.01%, w/v, naphthol AS-MX phosphate, 0.06%, w/v, Fast Red Violet LB salt and 1%, v/v, N,N- dimethylformamide in staining buffer, pH:5; 40 mM sodium acetate and 10 mM disodium tartrate dehydrate). TRAP+ cells were observed and captured by light microscopy [20].

To measure TRAP enzyme activity, scaffolds were lyzed with 200 μL of 0.1% (v/v) Triton X-100, then sonicated at 4 °C for 10 min [21]. Samples were centrifuged at 13,000 rpm for 3 min. Afterwards, 20 μL of cell lysate was incubated with 180 μL TRAP solution (2.5 mM pNPP, 0.1 M sodium acetate buffer, pH 5.8, 0.2 M KCl, 10 mM disodium tartrate, 1 mM ascorbic acid and 100 μM FeCl_3_) for 30 min at 37 °C. Optical density resulted from the conversion of pNPP to p-nitrophenol was detected at 405 nm by plate-reader [22].

#### Real-time polymerase chain reaction (PCR)

The expression levels of TRAP, NFATC1 and cathepsin K in osteoclast differentiation of RAW 264.7 cells cultured on chitosan/HAp scaffolds were assessed via real-time PCR. Expression levels of these genes were quantified using a Light Cycler^®^ Nano (Roche, Switzerland) and they were normalized to housekeeping gene *β*-actin. The data were calculated as fold change using the 2^−(ΔΔCt)^ method. The unloaded group was selected as the control and the relative gene expression was calculated as 1. Gene expression of the other groups was calculated comparatively to the control group. The sequences of the primers used in this experiment were given in Table 1.

**Table 1:** The primer sequences used in this study

Genes	Primers
*β*-actin	
Forward	5′-GTGCTATGTTGCCCTAGACTTCG-3′
Reverse	5′-GATGCCACAGGATTCCATACCC-3′
TRAP	
Forward	5′-AGATTTGTGGCTGTGGGCGA-3′
Reverse	5′-CTGCACGGTTCTGGCGATCT-3′
NFATC1	
Forward	5′-GGGTCAGTGTGACCGAAGAT-3′
Reverse	5′-GGAAGTCAGAAGTGGGTGGA-3′
Cathepsin K	
Forward	5′-CACCCAGTGGGAGCTATGGAA-3′
Reverse	5′-GCCTCCAGGTTATGGGCAGA-3′

#### SEM imaging

SEM imaging was performed for observing the morphology, attachment and spreading of RAW 264.7 cells grown on chitosan/HAp scaffolds. At the end of day 5, culture medium was removed and samples were washed twice with PBS. Then, each scaffold was treated with 500 μL of 2.5% (v/v) glutaraldehyde and kept in dark for 30 min for cell fixation. Samples were washed with PBS and kept at 4 °C. After that, they were dehydrated in ethanol series (30, 50, 70, 90 and 100%) for about 2 min at each concentration. The samples were incubated for 5 min in HMDS and air-dried overnight. Finally, they were coated with gold-palladium and SEM imaging was performed at different magnifications.

### Statistical analysis

The experiments were performed in triplicate, and all data were expressed as mean ± standard deviation. Statistical analysis was carried out by GraphPad InStat software (GraphPad, USA). One-way ANOVA was used to determine significant differences among the groups and *p < 0.05, **p < 0.01, and ***p < 0.001 represent statistically significant, very significant, and extremely significant values, respectively; whereas *p* > 0.05 represents no statistically significant values.

## Results and discussion

### Preparation and characterization of chitosan/HAp scaffolds

Chitosan/HAp composite scaffolds were prepared by freeze-drying method; in which chitosan/HAp solution was frozen and freeze-dried to remove ice crystals formed during freezing and to obtain the porous and interconnected structure [23]. HAp, the major inorganic component of bone tissue, was integrated into chitosan scaffolds since it has increased the biocompatibility of composite scaffolds than that in pure chitosan as demonstrated in previous studies [24]. Composite scaffolds were then stabilized by ethanol to prevent rapid swelling and ultimate dissolution.

**Fig. 1:**
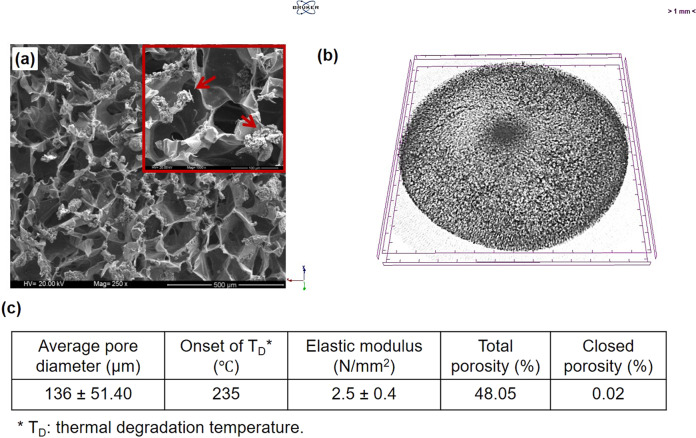
**a** SEM photograph of chitosan/HAp scaffolds (HAp beads were labeled with red and scale bars represent 500 µm for big image and 100 µm for the image given on the top right-hand corner), **b** μ-CT image of chitosan/HAp scaffold, **c** some basic properties of chitosan/HAp scaffolds

The morphological characterization of chitosan/HAp scaffolds was carried out by SEM. The porous and interconnected structure of chitosan/HAp composite scaffold is visible in Fig. 1a. The homogenous distribution of HAp beads within the chitosan matrix was observed in the same figure. The 3D microstructure of scaffolds was also visualized by micro-CT imaging. The porosity and interconnected pore spaces appear in Fig. 1b. In addition, some basic properties of the scaffolds were summarized in Fig. 1c. The relatively large pore size of the scaffolds (100–200 µm) facilitates the accommodation of cells onto scaffolds by offering high surface area and helps the transport of nutrient through the scaffold. Besides, the interconnected structure of the scaffolds offers cell infiltration into the structure [25, 26]. The scaffold is also thermally stable after stabilization with ethanol. The elastic modulus of the scaffolds was acceptable when compared to the ultimate compressive strength of trabecular bone (0.22–10.44 N/mm^2^).

### Release profiles of MEL and BMP-2 from chitosan/HAp scaffolds

**Fig. 2:**
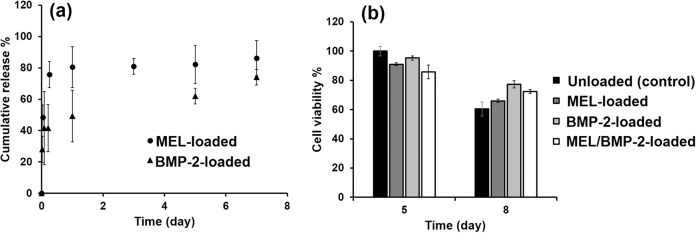
**a** Cumulative release profiles of MEL and BMP-2 from chitosan/HAp scaffolds, **b** viability of RAW 264.7 cells proliferated on blank, MEL-loaded, BMP-2-loaded and MEL/BMP-2-loaded chitosan/HAp scaffolds

MEL and BMP-2 were embedded into chitosan/HAp scaffolds with final concentrations of 300 ng/mL and 800 µM, respectively. Investigation of MEL and BMP-2 release profiles from MEL-loaded or BMP-2-loaded chitosan/HAp scaffolds up to 7 days revealed that, about 50% of MEL and 30% of BMP-2 were released, respectively, during the first hour with burst effect (Fig. 2a). At the end of day 7, ~86% and 75% of MEL and BMP-2 were released, respectively. The rapid release of MEL and BMP-2 from chitosan/HAp scaffolds was attributed to the large pore size of scaffolds (~136 µm). Since MEL is a smaller molecule than BMP-2, the release rate is higher than that of BMP-2.

### Cell culture studies

The effects of different concentrations of BMP-2 on osteoclast precursors cultured with either RANKL or RANKL/MCSF were investigated in the literature. The addition of 30 ng/mL BMP-2 was found to be a direct stimulant of the differentiation of bone marrow macrophages (BMM) cultured with 30 ng/mL RANKL [8]. However, other studies have demonstrated that the differentiation of RAW 264.7 cells pretreated with 30 ng/mL RANKL was not enhanced by adding 50 ng/mL BMP-2 as no significant differences were detected in the expression of osteoclast marker genes or in TRAP staining when compared to BMP-2-free group [27]. Another study also showed that the exposure of either BMM cells or RAW 264.7 cells to 100 ng/mL of BMP-2 was not sufficient to stimulate their differentiation to osteoclasts [28]. On the other hand, differentiation of BMM cells cultured with 10 ng/mL RANKL and 10 ng/mL MCSF was stimulated differentiation when 200 ng/mL BMP-2 was added [29]. Similarly, treating BMM cells with 300 ng/mL BMP-2 noticeably enhanced their differentiation to osteoclasts in the presence of 100 ng/mL RANKL and 100 ng/mL MCSF [9]. In this regard, it was suggested to use 300 ng/mL of BMP-2 to study its effect on the differentiation of RAW 264.7 cells cultured with 10 ng/mL RANKL and 10 ng/mL MCSF.

The inhibitory effect of MEL at its pharmacological concentration (1–100 µM) was investigated on BMMs cultured in medium supplemented with 20 ng/mL MCSF and 50 ng/mL RANKL. Results of TRAP staining and RT-PCR showed that MEL inhibited the osteoclastogenesis of BMMs in dose-dependent manner [30]. However, MEL concentration of 100 µM failed to inhibit the differentiation of RAW 264.7 cells to osteoclasts cultured with 4 ng/mL RANKL as TRAP-stained cells were not detected and expression of marker genes was not decreased compared to control group [31]. In another study, it was reported that MEL concentrations above 200 µM showed confirmed inhibitory effect on osteoclastogenesis [32]. Besides, we have demonstrated in our previous work that, MEL inhibited the differentiation of RAW 264.7 cells cultured with 10 ng/mL RANKL and 10 ng/mL MCSF in dose-dependent manner and 800 µM was selected as the most efficient anti-osteoclastogenic dose [18].

#### Cell viability

MTT assay was carried out to evaluate the proliferation of RAW 264.7 cells on blank, MEL-loaded, BMP-2-loaded and MEL/BMP-2-loaded chitosan/HAp scaffolds at day 5 and 8 of the culture. As observed in Fig. 2b, no statistical differences were detected among groups at days 5 and 8 suggesting that the cells in all groups nearly maintained their viability at the same level and the selected doses of loaded factors had no toxic effect on the cells. These findings are in accordance with literature as many experiments were performed to study the effect of BMP-2 on osteoclast formation using doses of 100 or 300 ng/mL and no cytotoxic effect was detected when they were used [9, 28]. In similar manner, the cytotoxic effect of MEL on RAW 264.7 cells was investigated in the range of 250–2000 µM and MTT results showed no decrease in cell viability [33].

#### Effect of MEL-loaded and BMP-2-loaded scaffolds on the differentiation of RAW 264.7 cells

**Fig. 3:**
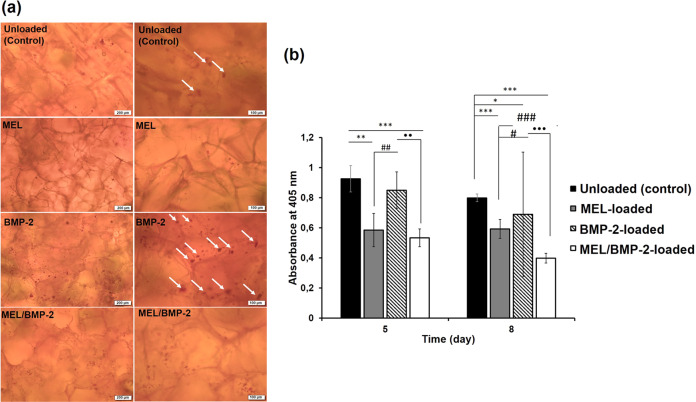
**a** TRAP staining of RAW 264.7 grown on unloaded and loaded chitosan/HAp scaffolds at day 8 at different magnifications, **b** TRAP activity. (Statistical differences when blank scaffolds are control: *p < 0.05, **p < 0.01, ***p < 0.001, when MEL-loaded scaffolds are control: ^#^*p* < 0.05, ^##^*p* < 0.01, ^###^*p* < 0.001, when BMP-2-loaded scaffolds are control: *p < 0.05, **p < 0.01, ***p < 0.001)

Differentiation of RAW 264.7 cells towards osteoclasts is mediated by RANKL and MCSF as previously reported [34]. In our study, we used RANKL and MCSF at low dose of 10 ng/mL for both of them to stimulate osteoclast formation. Besides, MEL and BMP-2 were loaded separately and together to study their single and synergistic effects on osteoclastogenesis. TRAP activity, TRAP staining and expression of marker genes were investigated to evaluate the differentiation of RAW 264.7 cells. As shown in Fig. 3a, number of TRAP-stained cells in MEL-loaded scaffolds was lower and their number in BMP-2-loaded scaffolds was higher than that of control group. In addition, number of stained cells in MEL/BMP-2-loaded scaffolds was less than that of BMP-2-loaded scaffolds. These results suggest that, MEL inhibited osteoclastogenesis of RAW 264.7 cells and their BMP-2-mediated differentiation. Osteoclast differentiation was also evaluated by TRAP activity (Fig. 3b). It was observed that there were no significant differences between unloaded control group and BMP-2-loaded group (*p* > 0.05). However, TRAP activity in MEL/BMP-2-loaded scaffolds was lower than that of BMP-2-loaded scaffolds at day 5 (**p < 0.01) and day 8 (***p < 0.001).

**Fig. 4:**
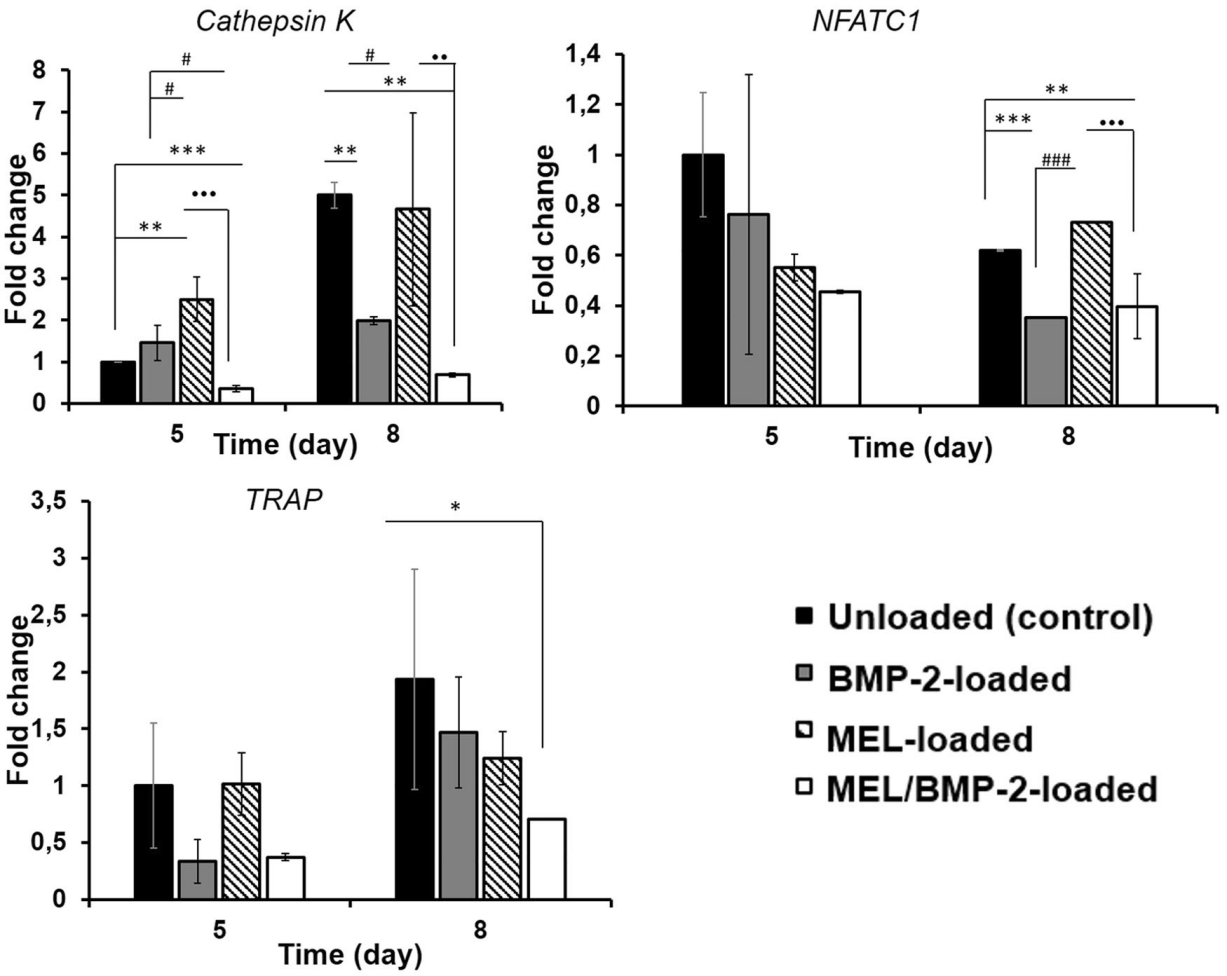
Gene expressions of RAW 264.7 cells grown on blank and MEL and/or BMP-2 loaded scaffolds, **a** cathepsin K gene, **b** NFATC1 gene and **c** TRAP gene. (Statistical differences when blank scaffolds are control: *p < 0.05, **p < 0.01, ***p < 0.001, when MEL-loaded scaffolds are control: ^#^*p* < 0.05, ^##^*p* < 0.01, ^###^*p* < 0.001, when BMP-2-loaded scaffolds are control: *p < 0.05, **p < 0.01, ***p < 0.001)

To further confirm the synergic effect of MEL and BMP-2, the expression of osteoclast marker genes including cathepsin K, NFATC1 and TRAP were examined as elucidated in Fig. 4. The expression of all mentioned genes significantly decreased in MEL/BMP-2-loaded scaffolds than that of control or BMP-2-loaded scaffolds inspite of the increase of the expression of only cathepsin K gene in BMP-2-loaded scaffolds at day 5 comparing to control group (**p < 0.01). These results are consistent with results obtained in previous studies reported in the literature. The number of TRAP+ cells increased when bone marrow cells were exposed to BMP-2 concentration of 300 ng/mL in the presence of RANKL and MCSF [9]. Similarly, the expression of NFATC1 and TRAP genes in bone marrow cells increased significantly when cells were treated with 30 ng/mL BMP-2 in the presence of suboptimal RANKL concentration (30 ng/mL) [8]. Unlike BMP-2, the development of TRAP+ osteoclasts decreased in dose-dependent manner after the exposure to 0–1 mM MEL concentrations in the presence of RANKL and MCSF [16]. Many studies in literature demonstrated that, anabolic factors can be used not only for the enhancement of new bone formation but also to prevent or reduce aggressive bone resorption caused by the activation of osteoclasts by implant materials or osteogenic factors used in the treatment of bone defects. One of the studies has used MEL to inhibit titanium-induced bone resorption in vivo [16]. In like manner, alendronate was used to attenuate bone resorption caused by BMP-2 high dose [12]. In our study, we aimed to inhibit BMP-2-mediated osteoclastogenesis by MEL and RT-PCR results showed that MEL/BMP-2-loaded scaffolds had inhibitory effect on the differentiation of RAW 264.7 cells.

#### Morphological analysis

**Fig. 5:**
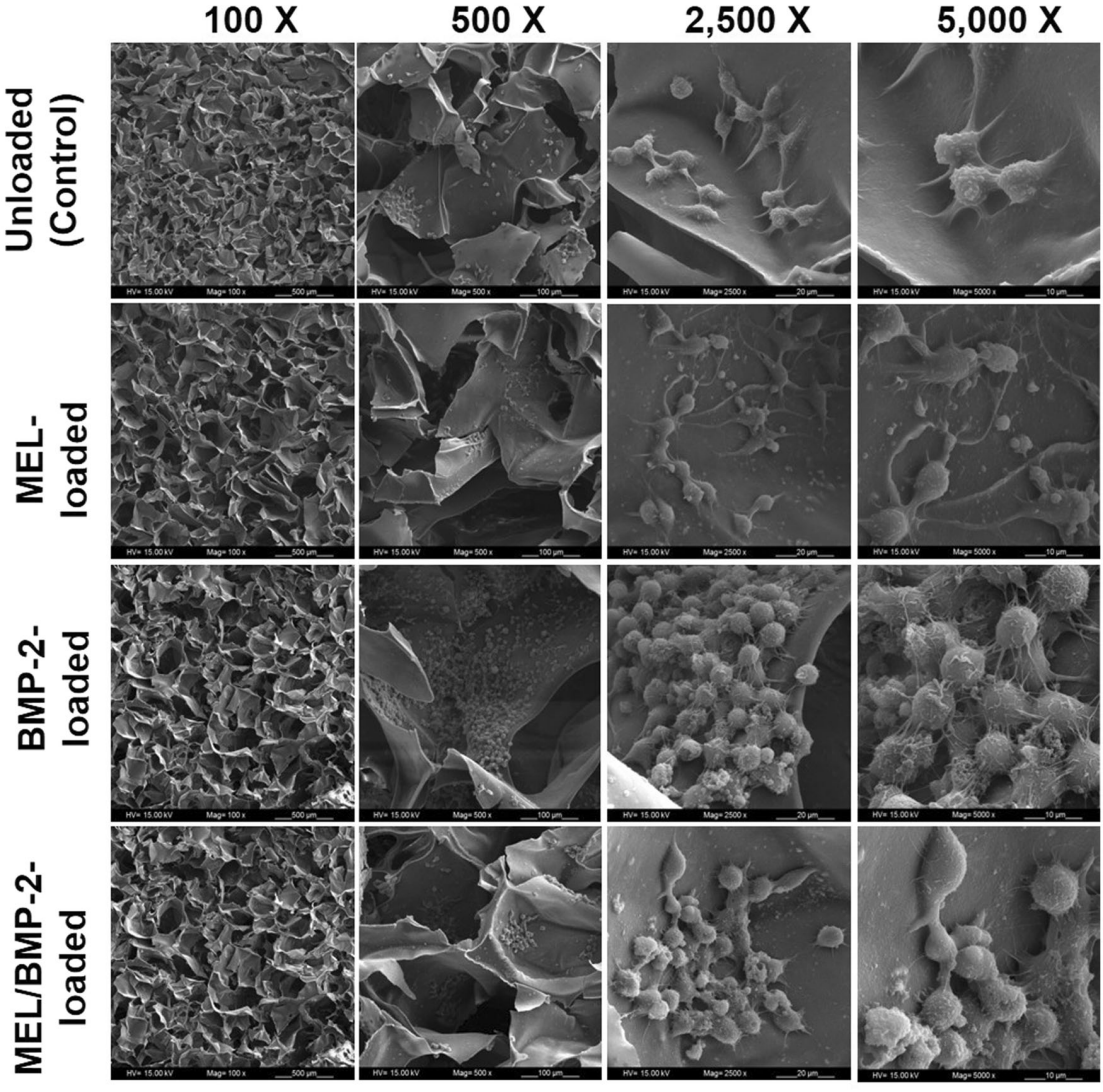
SEM images of RAW 264.7 cells grown on unloaded (control) and MEL and/or BMP-2-loaded chitosan/HAp scaffolds on day 5 at different magnifications

The morphology, size of cells and interactions between RAW 264.7 cells and scaffold were investigated by SEM analysis. As observed in Fig. 5, cells were attached and spread within scaffolds and the interactions with scaffold and with neighbor cells were maintained suggesting that MEL and/or BMP-2 loaded scaffolds were supportive for cell accommodation. Fig. 5 also shows that the number of cells was high and they had large sizes and rounded morphologies in BMP-2-loaded scaffolds when compared to other groups indicating that BMP-2 enhanced proliferation and differentiation of RAW 264.7 cells toward osteoclasts as they are distinguished with their large size. Besides, the number of cells was lower and the sizes of cells were varied from small to large in MEL/BMP-2-loaded scaffolds indicating that the BMP-2-mediated osteoclast formation was attenuated by the inhibitory effect of MEL.

## Conclusion

In this study, we established a co-delivery 3D system by loading of MEL and BMP-2 into chitosan/HAp scaffolds. The construct showed inhibitory properties on osteoclast differentiation of RAW 264.7 cells as demonstrated in TRAP activity, TRAP staining, expression of marker genes and SEM imaging. In addition, MEL successfully attenuated osteoclast differentiation mediated by BMP-2 high dose as appeared in TRAP staining, SEM and expression of cathepsin K gene. Taking together, we suggest that the use of MEL and BMP-2 loaded scaffolds would be considered for bone regeneration, especially when osteoclast resorption is threatening, with their osteogenic cell-differentiating and osteoclast-suppressing properties.
